# Malnutrition in Hospitalized Old Patients: Screening and Diagnosis, Clinical Outcomes, and Management

**DOI:** 10.3390/nu14040910

**Published:** 2022-02-21

**Authors:** Francesco Bellanti, Aurelio lo Buglio, Stefano Quiete, Gianluigi Vendemiale

**Affiliations:** Department of Medical and Surgical Sciences, University of Foggia, Viale Pinto 1, 71122 Foggia, Italy; francesco.bellanti@unifg.it (F.B.); aurelio.lobuglio@unifg.it (A.l.B.); stefanoquiete@gmail.com (S.Q.)

**Keywords:** malnutrition, hospitalized, aging

## Abstract

Malnutrition in hospitalized patients heavily affects several clinical outcomes. The prevalence of malnutrition increases with age, comorbidities, and intensity of care in up to 90% of old populations. However, malnutrition frequently remains underdiagnosed and undertreated in the hospital. Thus, an accurate screening to identify patients at risk of malnutrition or malnourishment is determinant to elaborate a personal nutritional intervention. Several definitions of malnutrition were proposed in the last years, affecting the real frequency of nutritional disorders and the timing of intervention. Diagnosis of malnutrition needs a complete nutritional assessment, which is often challenging to perform during a hospital stay. For this purpose, various screening tools were proposed, allowing patients to be stratified according to the risk of malnutrition. The present review aims to summarize the actual evidence in terms of diagnosis, association with clinical outcomes, and management of malnutrition in a hospital setting.

## 1. Introduction

Malnutrition in hospitalized patients represents a heavy healthcare burden worldwide [1]. Indeed, malnutrition in hospitalized patients worsens both prognosis and quality of life by increasing mortality, morbidity, and infection rate, extending the hospital stay, reducing the response to medical treatment, and increasing both the re-hospitalization rate and health expenditure [1,2,3,4,5].

The increase in malnutrition-related diseases in people with multiple comorbidities is a growing health concern, and it is strictly related to both the aging of the general population and the improvement in healthcare [2,6,7]. Of note, this population group more often needs hospitalization [2]. Between 20 and 50% of patients are present with malnutrition before hospital admission [5,8]. Of note, 49% of malnourished patients that are hospitalized for more than a week maintain or face a deterioration of their previous nutritional status [3]. Moreover, about a third of patients with a preserved nutritional status before hospital admission will develop malnutrition during hospital stay [5].

Several factors contribute to the worsening of nutritional status during hospitalization: illness-related loss of appetite, fasting for diagnostic procedures, drug-related side effects, diseases that compromise the regular functioning of the digestive system, and the poor management of patient nutrition [6].

Despite the relevance and the prevalence of the problem, malnutrition frequently remains underdiagnosed and undertreated. Diagnosis of malnutrition needs a complete nutritional assessment, which is often challenging to perform during a hospital stay [1,2]. Consequently, the real prevalence of malnutrition is not well-established, owing to the absence of a broadly accepted clinical definition [9,10].

Specific identification and optimal management of this clinical condition could improve the prognosis of malnourished patients, reducing both the length of the hospital stay and costs related to hospitalization [10].

## 2. Definition of Malnutrition

Malnutrition is a global health problem affecting more than a billion people of any age group [6,11]. Despite its worldwide diffusion, a universal definition of malnutrition is not yet accepted, even because the definition and the diagnostic criteria for malnutrition have changed over time [12].

The European Society for Clinical Nutrition and Metabolism (ESPEN) defines malnutrition as “a state resulting from lack of intake or uptake of nutrition that leads to altered body composition (decreased fat-free mass) and body cell mass, leading to diminished physical and mental function and impaired clinical outcome from disease” [13]. The American Society of Parenteral and Enteral Nutrition (ASPEN) and the Academy of Nutrition and Dietetics (Academy) suggest etiologic-based definitions that consider both time and degree of inflammatory response in acute or chronic illness/injury [14]. Considering its clinical and pathophysiological heterogeneity, the term “malnutrition” includes three major groups of conditions: (1) undernutrition, which includes stunting (low height-for-age), underweight (low weight-for-age), and wasting (low weight-for-height); (2) micronutrient-related malnutrition, which includes micronutrient excess or deficiencies (lack of important vitamins and minerals); (3) overnutrition, overweight, obesity, and diet-related non-communicable diseases (such as stroke, heart disease, diabetes, and cancer) [15]. 

Both ESPEN and ASPEN identified similar criteria for the diagnosis of malnutrition but with different clinical indicators [16,17]. Following the ESPEN recommendations, subjects at risk of malnutrition should be recognized by validated screening tool criteria, further assessed, and treated accordingly (Table 1). Two options were proposed for the diagnosis of malnutrition: the presence of a body mass index (BMI) <18.5 kg/m^2^, or the combined presence of unintentional weight loss (defined as a loss >10% than usual weight irrespective of time or a loss >5% over three months) associated to at least one of the following: reduced BMI (BMI <20 kg/m^2^ if the patient is younger than 70 years old, or BMI <22 kg/m^2^ if the patient is older than 70 years old), or low-fat free mass index (FFMI, <17 and <15 kg/m^2^ in males and females, respectively) [18].

ASPEN identifies six criteria, but at least two of them are required to diagnose malnutrition (Table 2): (1) low energy intake, (2) weight loss, (3) loss of muscle mass, (4) loss of subcutaneous fat, (5) fluid retention, and (6) reduced handgrip strength [14].

The proposed Global Leader Initiative on Malnutrition (GLIM) criteria for the diagnosis of malnutrition include: (1) unintentional weight loss, (2) low BMI, (3) and decreased muscle mass as phenotypic criteria, and (4) impaired food intake or assimilation and (5) burden of disease/inflammation as etiologic criteria (Table 3) [19]. The diagnosis of malnutrition needs at least one phenotypic and one etiological criterion. Lastly, GLIM consensus proposed phenotypic metrics for grading malnutrition severity as moderate (stage 1) and severe (stage 2) [19].

## 3. Screening and Assessment of Malnutrition in the Acute Setting

Malnutrition in a hospital setting is a costly, morbid, potentially preventable, and treatable issue. Even though malnutrition is a well-known determinant of several serious complications, hospitalized patients are not regularly screened for nutritional status at hospital admission and are seldom diagnosed if malnutrition occurs during hospital stay [2,20]. Nutritional screening tools in a hospital setting should be easy and quick to use, validated and suitable to be applied for bedside assessment, and highly effective in identifying individuals at risk of malnutrition [21,22]. The Nutrition Risk screening-2002 (NRS-2002) is one of the nutritional screening tools recommended by ESPEN. NRS-2000 was validated in adult inpatients and considers weight loss, weekly reduction of food intake, worsening of general conditions, disease severity, and age. Low risk of malnutrition is defined by a score <3, while patients with a score ≥3 are considered at medium/high risk of malnutrition. This tool was created to identify patients who should benefit of a nutritional intervention [2,6,17,23]. The malnutrition universal screening tool (MUST) is a further useful tool included in the ESPEN guidelines, validated in adult patients, and recommended in hospital, community, and other care settings. The MUST score includes three clinical parameters: weight loss, BMI, and reduction of food-intake for at least five days. Patients are defined at low risk of malnutrition if score is 0 and at medium risk if score is 1, while a score of 3 defines malnutrition [2,6,17]. In older people, ESPEN recommends using the Mini Nutritional Assessment (MNA) either in its full or short form (MNA-SF). MNA-SF is a valid alterative to MNA in hospitalized elderly patients. It evaluates 6 items from MNA: (1) reduction of food intake in the previous three months, (2) mobility, (3) acute disease or psychological stress, (4) neuropsychological problems, and (5) BMI or (6) calf circumference (CC). This tool identifies three categories of patients: those with a preserved nutritional status; those at risk of malnutrition, and those who are malnourished. The MNA is a moderately validated tool, not suitable for old patients that suffer from a severe cognitive decline [2,6,17].

In order to diagnose malnutrition, a 2-step approach has been recently recommended by the Global Leadership Initiative on Malnutrition (GLIM) in hospitalized patients: the first step consists of a screening within 24–48 h after hospitalization to identify subjects at risk of malnutrition using any validated screening tool; the second step includes the assessment for diagnosis and grading of severity in high-risk patients. Moreover, GLIM recommends to re-assess the nutritional status periodically during hospitalization [11]. 

A comparison among three nutritional screening tools (MUST, SGA and NRS-2002) in old hospitalized patients showed that MUST is the most sensitive, specific, and accurate to identify malnourished patients according to the new GLIM criteria for Malnutrition, despite being less rapid as compared to NRS-2002 and SGA [2]. Other validated nutritional screening tools include the Malnutrition Screening Tool (MST), which assesses weight loss and the decrease in food intake, and the Short Nutritional Assessment Questionnaire (SNAQ), which evaluates weight loss, appetite decrease, and the use of nutritional supplements (in the form of drinks or feeding tubes). Both nutritional screening tools are moderately validated and indicated for adult inpatients [6,17].

Nutritional screening should be included in a defined clinical protocol, followed by concrete interventions when needed [24]. A positive screening of malnutrition is followed by the assessment of the nutritional status. Indeed, this evaluation is performed if a patient is classified as at risk by a validated screening tool and by the assessment according to the new GLIM diagnostic criteria [2,11,19].

## 4. Epidemiology of Hospital Malnutrition

### 4.1. Prevalence of Malnutrition in Hospital Setting

**Figure 1:**
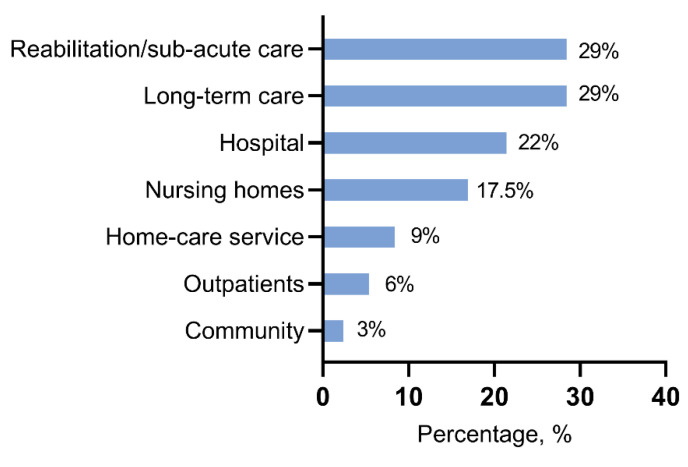
Prevalence of malnutrition based on the setting of care [27].

Malnutrition affects all stages of life, from children to old individuals, but prevalence differs depending on factors such as age, geography, social condition, or the presence of specific situations such as edentulia [6,25]. Nutritional status in hospitalized patients widely depends on the setting of care [26]. Indeed, the prevalence of malnutrition grows with the intensity care level, as shown in Figure 1 [27]. 

According to most studies, malnutrition prevalence in hospitalized patients ranges from 20% to 50%, according to the use of different diagnostic criteria and screening tools [2,7]. Among geriatric patients, the rate of poor nutritional status is higher as compared to younger, with a prevalence of up to 90% [7,28]. In our recent work, we found a prevalence of malnutrition of 46% among hospitalized old patients evaluated by the new GLIM diagnostic criteria [2]. In a recent prospective study on geriatric patients, malnutrition and risk of malnutrition were extremely prevalent between acutely ill medical patients, from admission to the Emergency Department up to four weeks after discharge [29]. Interestingly, hospitalized old patients do not show agreement between self-perceived and objective nutritional status [30].

### 4.2. Risk of Malnutrition during Hospitalization

About 60–65% of hospitalized patients experience a poorer nutritional status as compared to healthy individuals [31,32]. Indeed, hospitalization is associated with important changes in regular nutritional intake because of several factors, such as restricted timing of food provision, decreased appetite, adverse effects of medication, and prescribed periods of fasting [33]. A previous study demonstrated that, despite a protein provision of 1.0 g/Kg/day, the effective protein intake during hospitalization was lower in terms of reduced intake in both provided food and oral nutritional supplements [33]. Poor appetite, hospital meal refusal, and operation-related fasting are the most frequent causes of avoidable hospital-acquired malnutrition (HAM) [34]. Indeed, evidence indicates how the risk of malnutrition is higher when evaluated during hospitalization than assessed at hospital admission [35]. A prospective observational study demonstrated that decline in nutritional status and weight loss were significantly associated with protracted length of stay (LOS), independently of demographic features and disease severity [36]. On the other hand, malnutrition exerts a negative impact on LOS and several other clinical and economic outcomes [36,37]. A recent retrospective study reported a 1% incidence of malnutrition during hospitalization longer than 14 days [38]. Furthermore, HAM was significantly associated with extended LOS, cognitive impairment, pressure wound, or fall during hospital stay [38].

### 4.3. Risk Factors for Malnutrition in Hospitalized Patients

Several risk factors are described as associated with malnutrition in hospitalized patients [29]. Generally, these risk factors can be classified into two main groups: individual (physical and social) and organizational [39]. Old age, comorbidities, and polypharmacy are the most important physical risk factors for malnutrition [29,39]. 

Aging is associated with a higher risk of malnutrition because of several age-related changes able to affect nutritional statuses, such as deficit of physical activity, poor appetite, the feeling of unwantedness, or a sense of neglect. Furthermore, old age is characterized by a loss of taste, which can impact eating habits with negative consequences on health status [40]. Other important changes involve loss of bone density or skeletal muscle mass, with gain in body fat that can lead to osteoporosis, sarcopenia, or sarcopenic obesity [25,41]. In this scenario, malnourished old patients show a high risk of developing geriatric syndrome as compared to well-nourished, resulting in significant impairment of health status [42]. Cancer is highly associated with malnutrition in hospitalized patients. Malnutrition in cancer patients may depend on several mechanisms, including the tumor type, disease stage, side effects related to the treatment, and inadequate nutritional therapy [43,44]. Moreover, heart failure or diabetes mellitus are common diseases with a high prevalence of malnutrition and higher in-hospital mortality rates [45,46,47,48]. Polypharmacy is associated with malnutrition, especially proton pump inhibitors, anti-constipation, and antihypertensive drugs [49]. Malnutrition and polypharmacy are tightly related since an impairment of nutritional status induces the use of higher drug doses, creating a vicious cycle. Drugs can affect nutritional status through various mechanisms such as reduced appetite, decreased nutrient absorption, or adverse reactions [31,49]. 

Female sex is also associated with a higher risk of malnutrition due to several factors such as longer life expectancies than men or a higher probability to suffer from adverse economic and social circumstances in old age [50]. 

Low adherence to a Mediterranean diet is an important predictor of malnutrition in old patients [51,52]. 

Further factors include depression, low functional capacity, cognitive impairment, dysphagia, and eating-related problems [29,53]. Overall, the prevalence of eating difficulties during hospitalization was found to involve 46% of patients [54]. An important role in the risk of malnutrition is played by social factors such as low educational level and living alone [53]. In old patients, loneliness is recognized as an independent factor associated with poor nutritional status. Old people show a greater risk of reduced social relationships, isolation at home, and fewer opportunities to socialize with other people [55,56]. Nutritional status is further affected by marital status since unmarried subjects present with a higher risk of malnutrition [57,58]. 

Alcohol abuse, tobacco use, or socio-economic status are common independent risk factors of malnutrition [39,43,59,60]. Organizational factors play an important role in enhancing the risk of malnutrition, especially in hospitalized patients. Indeed, hospitalization is a risk factor itself for malnutrition [13,39]. Inadequate meal service, limited food choice, insufficient time to consume meals, and need to help for meal assumption are some of the principal risk factors to promote the decline in nutritional status during hospitalization [13]. Furthermore, malnutrition often remains unrecognized because nutrition screening is frequently underperformed in hospitalized patients [13,28]. In a European-wide survey, data show how only half of the hospital units reported routine use of nutrition screening [32].

## 5. Clinical Outcomes of Malnutrition in Hospitalized Patients

### 5.1. Malnutrition and Length of Stay

Malnutrition is mutually associated with several worse clinical outcomes [30]. However, this association can be influenced by different criteria used to define malnutrition [7]. LOS is negatively impacted by poor nutritional status, with consequent high costs and risk of complications. Evidence shows how LOS can increase by 40–70% in malnourished patients [7,37,61,62,63]. On the other hand, increase in LOS is associated with worse nutritional status during hospitalization [64].

### 5.2. Malnutrition, Falls, and Other Complications of Hospitalization

Malnourished old patients are at a higher risk of falls in hospitals when compared with those with preserved nutritional status. In particular, the risk of falls increases up to 8.4% and 6.2% in geriatric and internal medicine wards, respectively, and the global fall rate rises up to 31.6% up to 39.5% in patients ≥80 years [65]. Old patients with malnutrition and independent activities of daily living (ADL) were recently reported with a 2.7 higher risk of in-hospital falls [66]. In a 5-year observational study, malnourished patients showed an 8-times higher risk of harmful falls during hospitalization as compared to well-nourished patients [67]. Further, malnutrition is associated with increased risk for septic shock, acute kidney injury, stroke, and intubation [68]. Similar evidence was observed for higher risk of nosocomial infection in hospitalized geriatric patients with malnutrition [69]. Higher Nutritional Risk Screening 2002 (NRS-2002) score is an independent predictor of non-ventilator hospital-acquired pneumonia [70]. Malnutrition may also be determinant in the development of delirium in geriatric hospitalized patients [71]. 

### 5.3. Malnutrition, Muscle and Functional Impairment, and Quality of Life 

During hospitalization, malnutrition is associated with reduced physical activity and a higher prevalence of frailty. Hospitalized patients with malnutrition showed similar muscle strength to well-nourished patients at admission but a reduction in mean muscle strength values at the end of hospitalization [72]. However, a different study showed an impact of nutritional status on muscle strength at admission but no effect on muscle strength loss during hospitalization [73]. A significant reduction in mean mid-thigh muscle cross-sectional area (CSA) was found at follow-ups during hospitalization among malnourished patients with respect to baseline. On the other hand, hospitalization itself is an independent risk factor in the unfavorable change of muscle architecture parameters. Particularly, pennation angle undergoes reduction regardless of nutritional status [72,73]. Impairment in Activities of Daily Living (ADL) and in quality of life (QoL) is also associated with malnutrition [63]. Similarly, an association between poor nutritional state and lower Barthel index was demonstrated in hospitalized old patients [74]. Of note, hospitalized patients with malnutrition or at risk of malnutrition showed a weak recovery function in ADL [75].

### 5.4. Malnutrition, Hospital Readmission, and Mortality

Hospitalized malnourished patients show a higher readmission rate (up to 180 days post-discharge) or in-hospital death risk than well-nourished patients. Several studies report a higher 30-day readmission rate in patients with poor nutritional status as compared to well-nourished patients [76,77,78,79]. In fact, targeted intervention to improve nutritional status has been shown to reduce hospital readmission up to 77% [80,81]. Furthermore, poor nutritional status is associated with higher in-hospital mortality and post-discharge mortality rates, independently of gender and age [52,82]. An MNA score <17 resulted as an independent factor for in-hospital mortality [52]. Reduced consumption of hospital meals was associated with a higher risk for in-hospital mortality [83]. Of note, nutritional intervention during hospitalization in medical wards is associated with reduced in-hospital mortality and reduced 30-day readmission rates as compared to the absence of nutritional support [84]. Malnourished seriously ill patients also show a higher post-discharge mortality rate up to one year [85,86].

## 6. Management of Malnutrition in the Hospital

**Figure 2:**
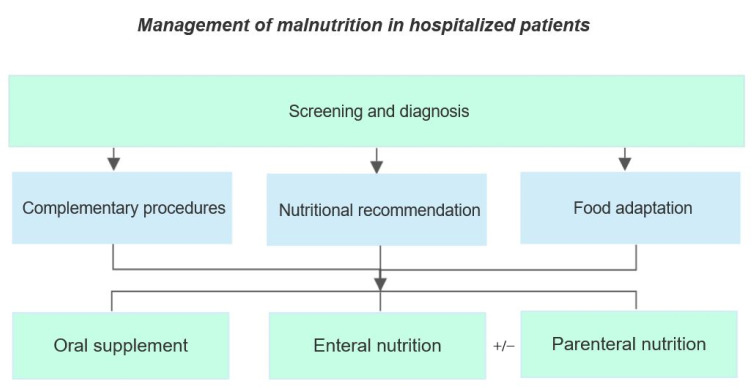
Intervention strategies to prevent or treat malnutrition according to ESPEN recommendations [27,89].

Despite a high prevalence of malnutrition, nutritional care is inadequate, and prevention measures are often not acquired [87]. Data from a recent survey show that 40% of medical/surgical staff and 58% of nursing staff are not able to diagnose malnutrition [88]. The therapeutic aims and approaches to malnutrition are similar both in young/adult and in old patients, even though the preservation of functional autonomy and quality of life in the latter group are more determinant than mortality [27]. Early diagnosis and consequent multidisciplinary approach are the main steps for the prevention and management of malnutrition in hospitalized patients [20,88]. According to the ESPEN guidelines for the management of malnutrition, different intervention strategies can be recognized to prevent or treat malnutrition (Figure 2). These strategies rely on (1) general recommendations (including screening of malnutrition), (2) supportive intervention, (3) nutritional counseling, (4) food modification, (5) oral food supplements, (6) enteral and parenteral nutrition [27,89].

### 6.1. Diagnosis

A universal method for the diagnosis of malnutrition in a hospital setting is not available. Screening tests are the first step to evaluate nutritional problems and consist in the administration of a rapid and easy tool such as the malnutrition universal screening (MUST), the nutritional risk screening-2002 (NRS-2002), or the mini nutritional assessment short-form (MNA-SF). In patients who are at risk of malnutrition or malnourishment, according to the screening tests, a comprehensive nutritional assessment should be performed [24,27]. The nutritional requirement to maintain global health status and dietary intake of energy, protein, electrolyte, mineral, micronutrients, fluids, and fibers should be guaranteed. Daily energy and protein intake in old people are estimated at 39 Kcal and at least 1 g protein per kg body weight, respectively [24,89]. Protein requirements can increase during hypercatabolic phases, such as acute illness and hospitalization, characterized by a high risk of muscle protein loss. Daily protein intake up to 2.5 g/kg was recommended for ICU patients, even though the ESPEN nutrition guidelines suggest 1.2–1.5 g/kg/day [90]. Nevertheless, several studies report that protein intake in hospitalized patients tends to be lower, up to 0.65 g/kg/day [33]. More than 60% of malnourished patients consumed an adequate protein intake starting from the fourth day after admission in one out of five Dutch hospitals [91]. Indicators for reduced food intake are old age, eating less during the previous week, bedridden condition, and hospitalization in internal medicine wards as compared to geriatrics or neurology wards [92]. Total energy expenditure (TEE) does not increase during hospitalization and is not influenced by inflammation [93]. Micronutrients play a determinant role in health status and quality of life because of their positive effect on every body system. Patients with multiple medical comorbidities may be at risk of micronutrient deficiency because of reduced intake or increased utilization. Furthermore, patients in enteral or parenteral nutrition can be at high risk of micronutrient deficiency and consequent malnutrition if meal replacements are not supplemented [94,95,96]. 

### 6.2. Complementary Procedures

Several conditions can be associated with a 35–40% reduced food consumption during hospitalization. Old people often experience impaired ability to eat and/or drink due to cognitive and functional limitations. Moreover, several chronic diseases such as diabetes mellitus or infections are significantly associated with inadequate nutritional behavior [33,89,97]. Barriers to food intake and mealtime/organizational issues are both factors susceptible to major preventive interventions [98]. Procedures based on service strategies to improve food intake show a promising positive effect on the nutritional state in hospitalized patients providing evidence to reduce both cost and readmission rate [99]. Increasing the daily frequency of food delivery in the hospital may increase protein intake at mealtimes and might satisfy the protein requirements of most patients [100]. An additional aspect worth consideration is related to the longer intragastric persistence of meals in old rather than young people, which is often the cause of compromised appetite. Thus, compounds or physical therapy aimed at favoring faster gastric emptying should be considered to manage malnutrition in old hospitalized patients [101]. Physical therapy may be of additional benefit to prevent loss of skeletal muscle mass in inactive patients, which is associated with lower energy needs and consequent appetite reduction [102,103,104].

### 6.3. Nutritional Recommendations 

ESPEN guidelines suggest that old people at risk of malnutrition or malnourishment and their caregivers should receive individualized nutritional counseling by dedicated dieticians or nutritionists to improve the awareness about nutrition and support healthy nutritional habits [27]. Patient-centered nutritional counseling is a recognized first-line approach to malnutrition management with proven benefits in several chronic conditions [105]. An automatic system based on Artificial Intelligence (AI) able to compare the established quantity of food before and after consumption through images is promising in detecting patients at high risk of malnutrition during hospitalization [106]. Modeling strategies as machine learning-based algorithms can be useful in the analysis of electronic health records to improve the diagnosis and management of patients at risk of malnutrition or malnourishment [107,108].

### 6.4. Food Adaptation

In old hospitalized patients, ESPEN guidelines recommend that food enrichment with natural ingredients (e.g., oil, eggs, cream, butter) or specific nutrient preparations (e.g., protein powder, maltodextrin) may improve both protein and energy intake with meals and beverages through consumption of similar food quantities [89]. Furthermore, corrections of carbohydrate, fat, protein and micronutrient intake can be required in particular clinical conditions or chronic diseases [109]. Snack and/or finger food between meals should be offered during hospitalization to increase dietary intake, taking into account circumstantial factors such as altered senses of taste, smell, or appetite, especially in old people [89,110]. Indeed, the gustatory decline does not induce old people to prefer strong flavors, but to consume more sweet and salty meals, while eating habits seem more influenced by psychological and social factors [40]. Although food-based fortification has shown helpful effects on calorie and protein intake, due to the limited number of participants and the poor quality of studies, further specific investigations are needed to provide reliable evidence [111]. 

### 6.5. Oral Supplements

Concentrated sources of nutrients that supplement a normal diet represent an option for the prevention and treatment of specific types of malnutrition. Oral supplementations can be represented by several elements or substances with a nutritional or physiological effect [109]. Several styles (milk, yogurt, juice, savory), formats (liquid, pudding, powder, pre-thickened), volumes, types (fiber-containing, high protein), energy densities (one to three kcal/mL), and flavors are available to match a wide range of needs and requirements [89]. Especially in old people, positive effects of high-energy ad high-protein oral supplementation were observed [112]. According to the ESPEN guidelines, oral food supplementation should provide at least 400 kcal with 30 g or more proteins per day [27].

### 6.6. Enteral and Parenteral Nutrition

Enteral (EN) and parenteral (PN) nutrition should be considered when patients are unable to meet energy and protein requirements according to diagnosis, clinical status, prognosis, and patient/family preferences. Conditions such as trauma, burns, and malignancy may require EN or PN to satisfy increased nutritional needs [113]. EN should start after a comprehensive patient evaluation which includes both risks and benefits if oral intake is absent or insufficient, or in patients moderately or severely malnourished [113]. In ICU patients, evidence suggests starting EN within the first 24–48 h from admission, preferring the orogastric or nasogastric tube. When the request for EN is more than four weeks, percutaneous access should be considered [114,115]. Obstinate vomiting or diarrhea, severe gastrointestinal (GI) malabsorption, GI ischemia, paralytic ileus, severe short bowel syndrome, or high output enterocutaneous fistula may represent contraindications to enteral feeding [113]. PN should be considered in patients with severe nutritional risk when EN is not achievable or after the first week of hospitalization if EN is not satisfactory [115]. Risks of overfeeding associated with the use of high-calorie PN, such as hyperglycemia and hypercapnia, frequently lead to less-caloric PN prescribing [116]. A recent ASPEN update reported that 46% of patients in EN and 40% of patients in PN were aged ≥65 years old, and the prevalence of malnutrition was higher compared to the prevalence of EN or PN use. These data may be explained by a missed coding of prescription, so that EN and PN may be improperly classified as food/medication or as consequences of shorter hospital length of stay [117].

A particular category of hospitalized patients with poor nutritional intake is represented by old people with advanced dementia. Both EN and PN have been discouraged in such patients because of increased risk of aspiration, gastrointestinal complications, fluid overload, infections, and bleeding [118]. EN or PN are indicated only in case of high risk of malnutrition and compromised swallowing, with possible aspiration pneumonia [119]. The decision of introducing EN or PN in patients with advanced dementia is challenging, so caregivers need complete information with a clear prognosis of the patient, being aware of the risks and benefits of artificial nutrition [120].

### 6.7. Effect of Nutritional Interventions in Hospital

A systematic review and meta-analysis reported that nutritional support was associated with up to 53% reduced risk of mortality rate and a 27% decrease in mean mortality risk [121]. Patients who benefited from nutritional support had a significant reduction in non-elective hospital readmission and higher protein/energy intake and weight gain, but no significant differences in infections, functional outcomes, and LOS [121]. Further work showed that a nutritional intervention was associated with a 25% decrease in LOS and a 35.7% reduction in infection rates in hospitalized patients with malnutrition or at risk of malnutrition [122]. Oral amino acid administration for seven days was associated with shorter LOS, a lower rate of post-discharge falls, and re-hospitalizations in old patients [123]. Moreover, preserved muscle strength and architecture and reduced circulating markers of oxidative stress were observed in hospitalized old patients supplemented with oral amino acids [123]. A recent meta-analysis, based on 29 randomized controlled trials, confirmed the positive effect of oral and enteral nutritional support, with a 30% of mortality reduction [124]. High protein strategies and long-term nutritional intervention were described as the most important predictors for the nutritional effect [124]. After discharge, nutritional intervention shows improvement in quality of life and physical function, lower LOS but no effect on readmission at six months [125]. EN and PN were also associated with lower LOS and mortality rates in hospitalized patients [126]. 

According to the results of a meta-analysis, supplementation with oral nutritional supplements (ONS) during and after a hospital stay resulted in a 16% decrease in hospital readmissions in old patients [127].

## 7. Conclusions and Future Perspectives

Nutritional status in hospitalized patients is determinant for several clinical outcomes. However, to date, screening to detect patients at risk of malnutrition or malnourishment is still poorly performed on admission and during hospitalization. Several rapid tools are validated in the hospitalized population, including old patients. The key to success in the prevention and management of malnutrition in hospitals is given by a multidisciplinary approach that identifies and treats the specific risk factors for each patient. Technology increasingly shows a potential positive role in approaching the diagnosis and treatment of malnutrition. Implementation of new strategies, such as the use of machine learning-based algorithms to analyze electronic health records or food analysis consumption by an automatic system based on AI, may represent a promising future approach to improve screening and management of hospitalized patients at risk of malnutrition or malnourished.

## Figures and Tables

**Table 1:** Diagnostic criteria for malnutrition according to the European Society of Clinical Nutrition and Metabolism [17].

1. Patients Classified as “at Risk” with Any Validated Risk Screening Tool	
2a Either:	2b Or:
BMI <18.5 kg/m^2^	Unintentional weight loss >10% indefinite of time, or >5% over the last 3 months,
	combined with either ‑ BMI <20 kg/m^2^ if <70 years of age, or 22 kg/m^2^ if ≥70 years of age Or ‑ FFMI <17 and 15 kg/m^2^ in men and women, respectively

BMI, Body Mass Index; FFMI, Fat-Free Mass Index.

**Table 2:** Diagnostic criteria for malnutrition in the context of acute illness or injury, according to the American Society of Parenteral and Enteral Nutrition [14].

Presence of at Least Two of the Following				
	Moderate		Severe	
Insufficient energy intake	<75% of estimated energy requirements for >7 days		≤50% of estimated energy requirements for ≥5 days	
Weight loss (from baseline)	%	Time	%	Time
	1–2 5 7.5	1 week 1 month 3 months	>2 >5 >7.5	1 week 1 month 3 months
Loss of muscle mass	Mild		Moderate	
Loss of subcutaneous fat	Mild		Moderate	
Fluid retention	Mild		Moderate to Severe	
Handgrip strength	No reduction		Reduced	

**Table 3:** Diagnostic criteria for malnutrition according to the Global Leader Initiative on Malnutrition [19].

Two-Step Approach	
First step: identify at-risk patients using any validated screening tool	
Second step: at least one Phenotypic and one Etiologic criterion are necessary to diagnosis.	
Phenotypic Criteria:	Etiologic Criteria:
Nonvolitional weight loss Low BMI Reduced muscle mass	Reduced food intake or assimilation
	Disease burden/inflammation

BMI, Body Mass Index.
